# The Chloroplast RNA Binding Protein CP31A Has a Preference for mRNAs Encoding the Subunits of the Chloroplast NAD(P)H Dehydrogenase Complex and Is Required for Their Accumulation

**DOI:** 10.3390/ijms21165633

**Published:** 2020-08-06

**Authors:** Benjamin Lenzen, Thilo Rühle, Marie-Kristin Lehniger, Ayako Okuzaki, Mathias Labs, Jose M. Muino, Uwe Ohler, Dario Leister, Christian Schmitz-Linneweber

**Affiliations:** 1 Molecular Genetics, Humboldt-University Berlin, 10115 Berlin, Germany; benjamin.lenzen@hu-berlin.de (B.L.); marie-kristin.lehniger@hu-berlin.de (M.-K.L.); okuzaki8@agr.tamagawa.ac.jp (A.O.); 2 Plant Molecular Biology, Department of Biology, Ludwig Maximilian University of Munich, 82152 Munich, Germany; thiloruehle@hotmail.com (T.R.); mathiaslabs@gmail.com (M.L.); leister@lmu.de (D.L.); 3 Computational Regulatory Genomics, Humboldt-University Berlin/Max Delbrück Centre for Molecular Medicine, 10115 Berlin, Germany; jose.muino@hu-berlin.de (J.M.M.); Uwe.Ohler@mdc-berlin.de (U.O.)

**Keywords:** chloroplast, RNA processing, RNA binding, NDH complex, RRM, organelle, *Arabidopsis thaliana*

## Abstract

Chloroplast RNA processing requires a large number of nuclear-encoded RNA binding proteins (RBPs) that are imported post-translationally into the organelle. Most of these RBPs are highly specific for one or few target RNAs. By contrast, members of the chloroplast ribonucleoprotein family (cpRNPs) have a wider RNA target range. We here present a quantitative analysis of RNA targets of the cpRNP CP31A using digestion-optimized RNA co-immunoprecipitation with deep sequencing (DO-RIP-seq). This identifies the mRNAs coding for subunits of the chloroplast NAD(P)H dehydrogenase (NDH) complex as main targets for CP31A. We demonstrate using whole-genome gene expression analysis and targeted RNA gel blot hybridization that the *ndh* mRNAs are all down-regulated in *cp31a* mutants. This diminishes the activity of the NDH complex. Our findings demonstrate how a chloroplast RNA binding protein can combine functionally related RNAs into one post-transcriptional operon.

## 1. Introduction

Chloroplasts contain genetic information that is essential for photosynthesis. The expression of this information is realized by a unique mixture of ancestral bacterial and derived eukaryotic features [1]. Chloroplast gene expression can be regulated on the transcriptional level [2,3], but post-transcriptional processes are of at least equal importance [4,5,6]. The abundance of chloroplast mRNAs changes in response to external triggers and functionally related RNAs can be combined into larger groups of transcripts [7,8]. Sigma-factors required for the specificity of the plastid-encoded RNA polymerase have been demonstrated to co-regulate functional classes of chloroplast genes at the level of transcription [9,10]. How co-regulation occurs on the post-transcriptional level, remains unknown.

One key evolutionary change in post-transcriptional processes between chloroplast and their bacterial ancestors are the vastly increased RNA half lives in the organelle. In bacteria, transcription and translation are usually directly coupled and mRNAs have short half-lives (in the range of minutes; [11]) In chloroplasts, the half-lives of mRNAs are long (in the range of hours) and untranslated RNAs accumulate in large amounts [12,13]. Important protein families responsible for RNA stability and thus transcript accumulation are pentatricopeptide repeat (PPR) proteins and chloroplast ribonucleoproteins (cpRNPs, [14,15]). The cpRNP protein family consists of ten members in *Arabidopsis thaliana* [15]. All cpRNPs are targeted to the chloroplast post-translationally, and dedicated import receptors appear to be responsible for their transport across the chloroplast envelope [16,17]. Genetic analyses have demonstrated that *Arabidopsis* CP31A supports RNA editing at multiple sites and modulates the stability of mRNAs [18,19]. Interestingly, temperature modulates the effects of the loss of CP31A. In the cold, the germination rate of null mutants is reduced and their newly emerging leaf tissue bleaches at 8 °C [19]. All analyzed proteins of the photosynthetic apparatus are reduced in this cold-treated, defective tissue. This was linked to multiple defects in RNA processing (e.g., RNA splicing, RNA editing, and intercistronic processing), but is likely primarily caused by the strongly reduced accumulation of multiple chloroplast mRNAs [19]. By contrast, under normal growth temperatures, the most strong effect of the lesion in *cp31a* was observed for the *ndhF* mRNA, which encodes a subunit of the NADH dehydrogenase-like complex or short NDH complex [18]. *ndhF* is hardly detectable by RNA gel blot hybridization in *cp31a* mutants [18] and an analysis of remaining degradation products suggested that CP31A helps to protect this mRNA against degradation from its 3′-end [19]. RNA co-immunoprecipitation analyses demonstrated that CP31A binds to the 3′-UTR of *ndhF* and is required for the generation of the *ndhF* 3′-terminus under normal growth conditions [19]. A transcriptome-wide survey of RNA targets using RNA co-immunoprecipitation and microarray analysis (RIP-chip) uncovered dozens of mRNAs co-precipitated with CP31A that were functionally assigned to the various chloroplast thylakoid membrane complexes and also, to the ribosome. By contrast, mature tRNAs and rRNAs were not among the identified RNA ligands. Thus, CP31A has a broad RNA target range. However, the minimal defects observed at normal growth temperatures suggests a hierarchy of functional relevance of CP31A at least under these conditions. We, therefore, set out to determine quantitatively, which are the main targets of CP31A and analyze in a genome-wide, quantitative fashion, which RNAs—next to the *ndhF* mRNA—are most strongly affected by the loss of CP31A.

## 2. Results

### 2.1. CP31A Has a Preference for Transcripts Encoding Subunits of the NDH Complex

Previous studies using microarrays for the detection of RNA targets of CP31A were not quantitative, amongst other reasons because probes used to detect the different RNAs differed in size and sequence composition and were unable to differentiate between the two strands of a gene [19]. Another potential bias was introduced by sample preparation. For the previous RIP-chips, chloroplasts were prepared prior to extract preparation. Given that this procedure takes hours and is performed at or below 4 °C, changes in the association of CP31A with RNAs could occur. We therefore established DO-RIP-Seq for chloroplasts, which uses formaldehyde cross-linking in flash-frozen leaf tissues (Supplementary Figure S1A). DO-RIP-Seq has been set up previously for HEK29 cells to quantify the binding site of an RBP at the whole-transcript target level, but has not been applied to a whole organism yet [20]. Here, wild-type (WT) *Arabidopsis* seedlings were grown for 14 days under normal growth conditions and then subjected to formaldehyde cross-linking and immunoprecipitation (IP); [19]. The tissue lysates were treated with a nuclease to yield RNA fragments protected by RNA binding proteins. Three biological replicates were performed. Successful recovery of CP31A in precipitates was verified by immunoblotting (Supplementary Figure S1B). DO-RIP-Seq libraries were prepared from input and pellet samples for all replicates (Supplementary Figure S1C). We tested the reproducibility of the DO-RIP-Seq experiments by calculating pairwise correlation coefficients across all samples (Supplementary Table S1). We found strong correlations among the input samples (average Pearson coefficient (R): 0.985), and the pellet samples from the IP (R: 0.999). This analysis demonstrates high reproducibility between biological replicates of our DO-RIP-Seq assay (the data are deposited under the GEO accession GSE152579).

**Figure 1:**
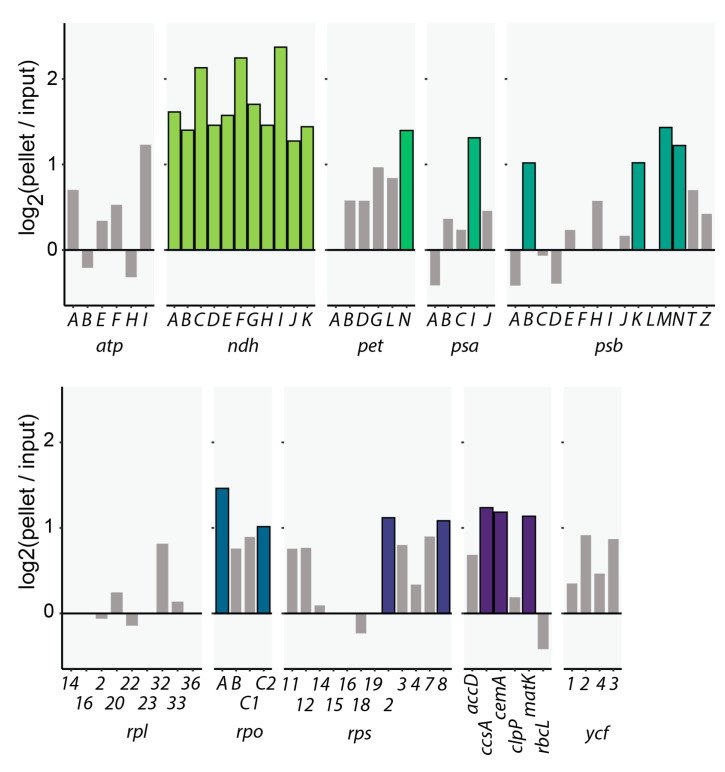
Target RNAs of CP31A identified by DO-RIP-Seq. The enrichment of RNAs in the pellet samples of CP31A over the respective input samples is shown for all protein coding chloroplast genes (based on the TPM normalized and log_2_-transformed read counts for every gene in every sample). Three biological replicates were used. Genes surpassing the cut-off criteria, namely enrichment of ≥log_2_(1) and ≤*p* 0.05 were considered targets. Results for non-target genes were greyed out.

For the analysis of DO-RIP-Seq reads, we mapped the reads to the chloroplast genome and counted reads for each annotated protein coding gene (including intronic regions), taking only reads into account that represent the functional strand (coding strand; according to NCBI acc. no. NC_000932.1). A finer mapping of binding sites turned out to be difficult since we obtained too few reads in the input library to allow calculation of enrichment ratios for shorter sequence stretches. The chloroplast genome is transcribed in overlapping polycistronic RNAs, making a definition of UTRs difficult. Nevertheless, most sequence elements important for RNA stability and translation are located proximal to the annotated regions. We therefore included for our analysis the 100 nt upstream and downstream of each gene, adding reads found here to the reads within the reading frames. Read counts were normalized using TPM, in order to account for gene length and library depth. A high pass filter was used to account for stochastic noise in low coverage genes, which excludes genes with less than 5 reads in any library from the analysis. We then calculated the enrichment of RNA in the pellet fraction of CP31A IPs versus the input fraction, averaging across the three replicates. Using an enrichment of at least two-fold and a significance cutoff for enrichment of *p* ≤ 0.05 (one-sided, paired *t*-test), we identified 24 target RNAs for CP31A (Figure 1). Remarkably, all 11 mRNAs encoding NDH subunits are significantly enriched (Figure 1) and of the 10 most enriched RNAs, 9 belong to the NDH complex (Supplementary Table S2). Gratifyingly and in line with our previous analyses, *ndhF* is the second most enriched mRNA in this analysis (Figure 1). We also performed immunoprecipitations with non-specific immunoglobulins (IgGs) as a negative control and sequenced the co-precipitated RNA. As expected, with one exception, no significantly enriched RNAs were found in this experiment (Supplementary Figure S1D). This demonstrates the specificity of RNA enrichment in CP31A precipitates.

### 2.2. NDH mRNAs Are Reduced in cp31a Null Mutants

**Figure 2:**
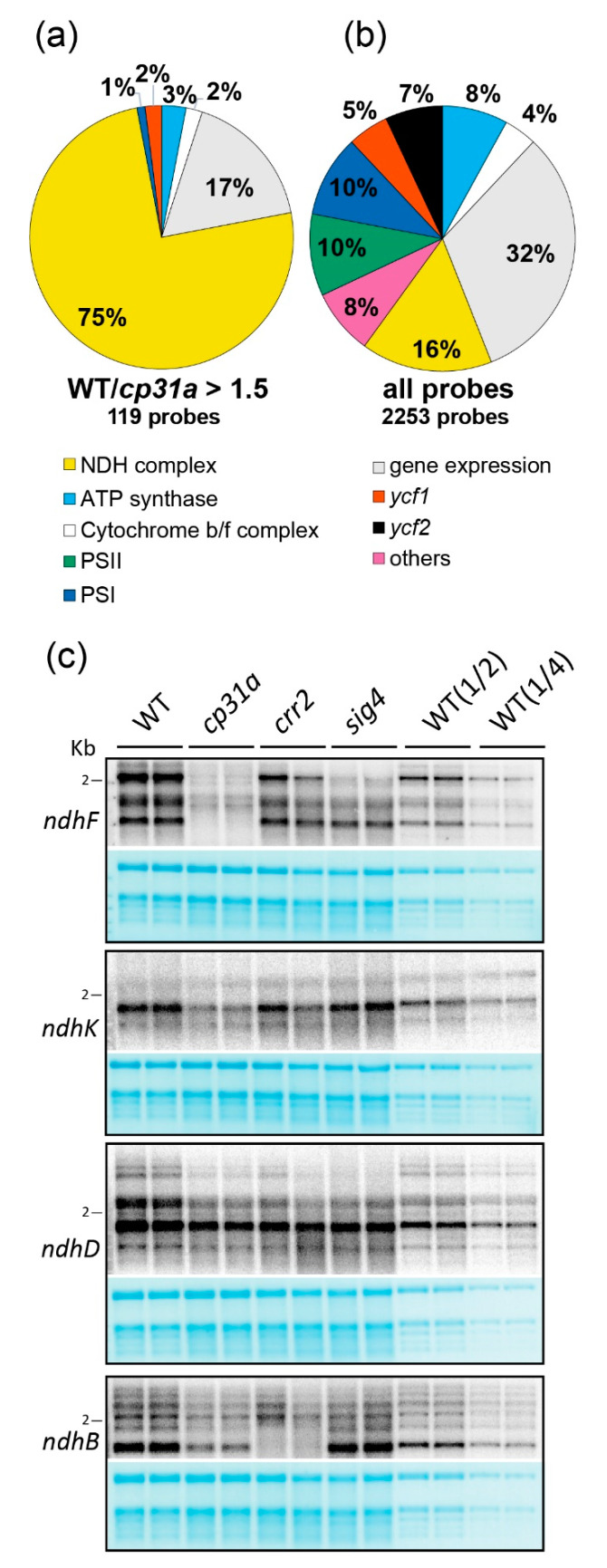
Analysis of RNA accumulation in *cp31a* mutants. (**a**) Summary of microarray analyses of 14 days old WT and *cp31a* mutant seedlings. Relative abundance of exon probes showing at least 1.5-fold stronger signals in WT versus *cp31a* mutants. Three replicate microarray hybridizations were analyzed and assigned to different gene categories. All results are presented in Supplementary Table S3. (**b**) Relative distribution of all exon probes on the microarray to different gene categories. (**c**) RNA gel blot analysis of 14 days old Arabidopsis seedlings. A quantity of 4 µg RNA from WT, *cp31a* mutants, and two control mutants (*crr2* and *sig4*) together with dilutions of WT samples (1/2 and 1/4) were probed with radiolabeled RNA probes against four different *ndh* genes. The resulting autoradiographs are always shown with the corresponding methylene blue stains of the membranes (below). The 2 kb marker band is shown as a reference.

We next investigated chloroplast RNA levels in *cp31a* versus WT samples using an oligonucleotide tiling array that represents the entire chloroplast genome of *Arabidopsis thaliana* in a tiling fashion. We scored only exon probes whose RNA levels were at least one-third lower in the mutant compared to WT. Of all exon probes in the array, 16% represent *ndh* sequences. Importantly, among exon probes whose signals were decreased in the mutants, 75% contained *ndh* sequences (Figure 2a,b, Supplementary Figure S2). No other functional category showed enrichment in this analysis. To confirm these results, we performed RNA gel-blot hybridization experiments for four *ndh* genes that represent the four *ndh* operons in the chloroplast genome (Figure 2c). We analyzed *cp31a* mutant RNAs alongside RNAs from seedlings with impairment in the expression of *SIGMA FACTOR 4* (*SIG4*), which is specifically required for the transcription of the *ndhF* mRNA [9], and *CHLORORESPIRATORY REDUCTION 2* (*CRR2*), which is required for the accumulation of monocistronic *ndhB* transcripts [21]. As expected, the control mutants showed specific defects for their known target RNAs (Figure 2c), while all other transcript patterns are identical to WT and in line with previous publications [19,21,22]. By contrast, the *ndhK* transcripts accumulated to normal levels in these two control lines, and the *ndhD* mRNA was only slightly decreased (Figure 2c). In contrast and consistent with our microarray results, the *cp31a* mutants displayed reductions in all four *ndh* transcripts analyzed. The decrease was most pronounced for *ndhF*, strong for *ndhK* and *ndhB*, and somewhat weaker for *ndhD* (Figure 2c). Collectively, these findings indicate that CP31A stabilizes mRNAs from all four *ndh* operons, suggesting that CP31A regulates *ndh* mRNAs as a group.

### 2.3. NDH Complex Activity Is Reduced, but Not Absent, in cp31a Mutants

**Figure 3:**
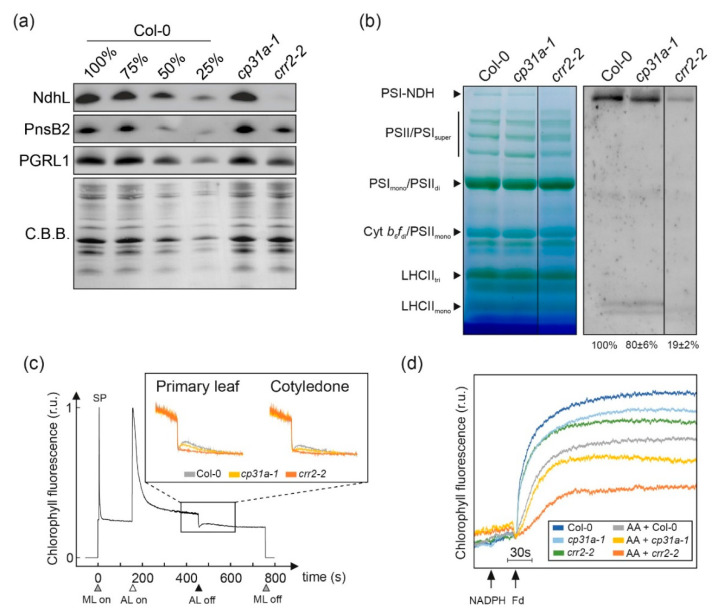
Analysis of NDH complex accumulation and activity in *cp31a* mutants. (**a**) Thylakoid membranes were isolated from wild-type (Col-0), *cp31a* and *crr2* plants. Proteins samples adjusted to 2.5 µg chlorophyll were size-fractionated on SDS-PAGEs, transferred to polyvinylidene difluoride membranes (PVDF) and probed with antibodies raised against NdhL, PnsB2 and PGRL1. As loading control, PVDF membranes were stained with Coomassie Brilliant Blue G-250 (C.B.B.). (**b**) Thylakoid membranes from wild-type (Col-0), *cp31a* and *crr2* plants corresponding to 80 µg chlorophyll were solubilized with 1% [*w/v*] *n*-dodecyl *β*-D-maltoside and fractionated by 4 to 12% BN-PAGE. The major protein complexes were assigned to individual bands as described [26]. Denatured protein complexes were then transferred to a PVDF membrane and probed with antibodies raised against the NdhT subunit of the NDH complex. NDH-PSI supercomplex amounts in *cp31a-1* and *crr2-2* were quantified relative to wild-type samples. Averages and standard deviations calculated from three technical replicates are shown below the immunodetection. (**c**) NDH activity of 4-week-old wild-type (Col-0), *cp31a* and *crr2* plants was assessed by chlorophyll fluorescence imaging analysis during a light-to-dark transition. Measurements were made with leaves or cotyledons of 2-week-old plants. Leaves were exposed to a saturating light flash (SP) to obtain maximum fluorescence (Fm) and then to low actinic light (AL) for 5 min. AL was then turned off and the subsequent transient rise in fluorescence ascribed to NDH activity was monitored using a pulse amplitude modulation chlorophyll fluorometer. The main panel shows the typical course of wild-type chlorophyll fluorescence under these conditions. Insets are magnified traces from the boxed area normalized to Fm. ML, measuring light. (**d**) Quantification of cyclic electron transport (CET) rates by measuring the increases in chlorophyll fluorescence in ruptured chloroplasts under low measuring light (1 µE/m^2^ s^−1^), after the addition of NADPH and Fd. Measurements were carried out with or without adding an inhibitor of the PGR5/PGRL1-dependent pathway of CET (Antimycin A = AA) to detect the activity of the alternative CET route via the NDH complex. Chlorophyll fluorescence was normalized to Fm, which was determined by application of an initial saturating light flash.

To analyze the effect of reduced *ndh* mRNAs on the NDH complex, we isolated thylakoid membranes from wild-type, *cp31a* mutants and *crr2-2* control mutants and subjected them to immunoblot analysis (Figure 3a). There were no working antibodies available to us for the chloroplast encoded subunits of the NDH complex, but is has been shown that the nuclear-encoded subunit NdhL is affected in mutants of the chloroplast-encoded subunits NdhB, D, and F [23]. However, both NdhL as well as a second nuclear-encoded NDH subunit, PnsB2, accumulate to almost WT levels in the *cp31a* mutant. In addition, disruption of CP31A does not affect protein levels of PGRL1 which is a component of the Antimycin A sensitive CET pathway (Figure 3a). We further examined PSI-NDH supercomplex integrity in *cp31a* (Figure 3b). To this end, we separated thylakoid complexes from wild-type, *cp31a* and *crr2-2* control mutants by blue native (BN)-PAGE, transferred denatured complexes to a PVDF membrane and immunodetected the NDH-complex subunit NdhT. NDH-PSI supercomplex formation is only mildly affected in *cp31a* (80 ± 6%), while it is clearly reduced in *crr2-2* (19 ± 2%) compared to the wild-type control. We next investigated the NDH activity by monitoring chlorophyll fluorescence during a light-to-dark transition. Such a light change leads to a transient increase in chlorophyll fluorescence, which is a result of the reduction of plastoquinone by the NDH complex. In mutants defective in NDH activity, this increase of fluorescence in the dark is lacking [24]. In *cp31a* mutants, no or only a minor reduction in the increase in fluorescence was observed, while the control mutant *crr2-2*, which is deficient in the NDH complex, is not showing any increase at all (Figure 3c). This measurement was carried out in primary leaves of 14 days old seedlings. By contrast, a marked decrease in post-illumination fluorescence is observed in cotyledons of the same plants (Figure 3c). Given that the analysis of post-illumination rise in fluorescence is not quantitative, it does not differentiate between different pathways to reduce plastoquinone and is thus in sum impractical to uncover more subtle differences in NDH activity, we decided to measure NDH activity with an additional method. In this assay, chlorophyll fluorescence is observed in purified and ruptured chloroplasts after the addition of NADPH and Fd under low measuring light [25]. The experiment is carried out either in the presence or the absence of antimycin A, an inhibitor of the alternative route of electrons around PSI via PGR5/PGRL1. We observed that the increase in chlorophyll fluorescence after addition of NADPH/Fd reached only a slightly lower plateau in *cp31a* compared with WT. However, after antimycin treatment the effect is much more pronounced (Figure 3d), although the plateau reached in *cp31a* mutants is still higher than in the *crr2-2* mutant (Figure 3d). This suggests that NDH activity is lowered in *cp31a* mutants relative to WT, but not fully absent. Moreover, the contribution of the NDH-dependent pathway to CET is minor under normal conditions (Shikanai et al., 1998) and the impact of CP31A disruption on the post-illumination fluorescence rise might be masked by the activity of the PGR5/PGRL1 pathway.

## 3. Discussion

### 3.1. CP31A Co-Regulates ndh Genes

During the evolution of chloroplasts from cyanobacterial ancestors, operon structures were disrupted, and operons were shuffled. Many chloroplast operons therefore include genes with different functions. The *ndh* genes, for example, are separated into four transcriptional units in *Arabidopsis* and are mixed with genes from other functional categories. This lack of conservation of operon structures suggests that transcriptional units are less important than other processes for *ndh* gene regulation in the chloroplast context, giving way to post-transcriptional processes. Translation plays an important role for regulation [27], but it remains unclear, how post-transcriptional co-regulation is achieved prior to translation. Our data lead us to propose that CP31A associates with all *ndh* mRNAs and that it prefers *ndh* mRNAs over any other transcripts. This was made possible by applying the DO-RIP-Seq technique to plants. We show that DO-RIP-Seq allows straight-forward quantification of RNA targets of plant RNA binding proteins. This complements the crosslinking and immunoprecipitation method (iCLIP) applied to plants previously, which allows base-resolution of RNA binding sites, but is also much more elaborate [28]. A caveat of this approach is that the cross-linking step may lead to fixation of interactions of CP31A with other proteins and thus it cannot be determined whether the identified RNA targets are directly or indirectly associated with CP31A. Given that cpRNPs have been demonstrated to bind RNA directly with their two canonical RNA recognition motifs [29,30], we assume that at least some of CP31A’s target RNAs in DO-RIP-Seq experiments are due to direct interactions. How CP31A identifies *ndh* transcripts is unclear at present. We did a survey of GC content and sequence motifs but could not identify anything that would separate the *ndh* genes from other chloroplast genes. Techniques to resolve the binding sites of CP31A could help to understand its preference for *ndh* mRNAs.

Importantly, the preference for *ndh* mRNAs is functionally relevant. *ndh* mRNAs are reduced in *cp31a* mutants more than any other gene class. Since the loss of CP31A does not impede transcription [19] and given that cpRNPs stabilize RNAs in vitro [31], we conclude that the protein is required for the stability of *ndh* mRNAs. Such a stabilizing role could be carried out in conjunction with other proteins, i.e., PPR proteins. An alternative explanation is that the loss of one RNA of the NDH complex has an indirect, hierarchical effect on the other *ndh* RNAs that yields the observed reduction in all *ndh* mRNAs. This phenomenon has been well described for hierarchical protein synthesis cascades in chloroplasts [32], but it has not been previously shown to be relevant at the level of mRNA synthesis or stability. Moreover, the likelihood of this scenario is weakened by our observation that the losses of *ndhF* or *ndhB* in the *sig4* or *crr2* mutants, respectively, were not followed by the loss of all *ndh* mRNAs. Taken together, our data indicate that CP31A serves to combine *ndh* mRNAs from different genomic loci into a post-transcriptional operon in the chloroplast. Similar post-transcriptional operons, or “RNA regulons”, have been well-described in fruit flies, budding yeast, and mammalian cells [33], but were unknown in chloroplasts. In most cases, these RNA operons function in the combined translation and/or stabilization of the participating RNAs [34,35,36]. Given the RNA-stabilizing roles of cpRNPs in general and of CP31A in particular, we propose that CP31A adjusts the stability of the *ndh* transcripts as a group.

### 3.2. CP31A Supports NDH Activity

The reduction of *ndh* mRNAs in *cp31a* mutants raises the question of the physiological consequences of CP31A activity. The NHD complex is known to contribute to electron routes from stromal sources into the plastoquinone pool and thus also supports cyclic electron flow around photosystem I [37]. The measurements of electron transport in *cp31a* mutants after light–dark shifts suggests that there is a decreased capacity to reduce the plastoquinone pool in the mutant. However, the reduction is at best mild and only clearly visible when measuring this effect in cotyledons. When we used Antimycin A to block the flow of electrons via the competing PGR5/PGRL1 shunt, we observed a clear reduction of cyclic electron flow in the *cp31a* mutants versus WT plants. These results indicate that the PGR5/PGRL1 route compensates for the reduced activity of the NDH complex in *cp31a* mutants. Importantly, the reduction in CET seen is not as pronounced as in *crr2* mutants. CRR2 mutants show a drastic reduction of the *ndhB* mRNA and were demonstrated previously to show a strong reduction in NDH complex accumulation and activity [21]. The comparatively mild reduction in *cp31a* mutant suggests that there is considerable residual NDH complex activity in these plants. This is in line with our observation that only a slight reduction of PSI-NDH supercomplex formation could be identified in BN PAGE gels. These results are at variance with our previous measurements that suggested a more drastic reduction of NDH activity in *cp31a* plants grown in the green house [18], while the plants analyzed here were raised in a phytotron. In general, different analyses of NDH complex mutants have yet to deliver a uniform picture of the function of the complex, its impact on electron transport, and other resulting phenotypes. This is particularly controversial when it comes to the role of the NDH complex under different stress conditions. There is, for example, an ongoing debate about the susceptibility of NDH complex mutants to strong light [38,39,40,41]. Moreover, NDH function can be activated by hydrogen peroxide [42] and is linked to the chloroplast redox regulation network via the NADPH-thioredoxin reductase NTRC [43]. Clearly, the function of the NDH complex differs depending on plant and leaf age, growth conditions, and which species is analyzed [44]. In this regard, it is noteworthy that we report here a lower activity of the NDH complex in cotyledons than in primary leaves of *cp31a* mutants. Cotyledons are not only older than primary leaves but are also physiologically and morphologically very different from primary leaves, all of which could underlie the bigger impact of the *cp31a* lesion on NDH complex activity in this tissue. In sum, we propose that differences in growth conditions and leaf age are behind the variance observed in NDH activity in *cp31a* mutants. Understanding the physiological relevance of CP31A for NDH complex activity under varying conditions will require further investigation.

## 4. Materials and Methods

### 4.1. Plant Growth

*Arabidopsis thaliana* Columbia-0, *cp31a-1* T-DNA insertion mutants [18] were grown on soil with a 16 h light/8 h dark cycle at 23 °C in a CLF growth cabinet at 120 µmol·m^−2^·s^−1^. For DO-RIP-seq experiments plants were grown on a soil/vermiculite 4:1 mixture at 21 °C for 14 days (normal conditions).

### 4.2. Immunoblot Analysis

Immunoblot analysis for the DO-RIP-seq experiments was performed as previously reported [19] with protein samples taken from the input, supernatant and pellet fraction of the co-immunoprecipitations.

### 4.3. DO-RIP-Seq Analysis

This protocol was built on previous efforts to identify quantitatively RNA species bound to an RBP in human cells [20,45]. *Arabidopsis thaliana* plants were harvested in triplicates after 14 days and flash-frozen in liquid nitrogen. The flash-frozen plant material was ground in liquid nitrogen. Between 250 to 350 mg plant material was suspended in 3 mL DO-RIP-seq lysis buffer containing formaldehyde (50 mM HEPES-KOH pH 8.0, 200 mM KCl, 5 mM MgCl_2_, 5 mM CaCl_2_, 0.5% Nonidet P-40, 0.5% Sodium deoxycholate, 1x cOmplete^TM^, EDTA-free Protease Inhibitor Cocktail (Roche), 1% formaldehyde) per 1 g plant material. Crosslinking was performed for 10 min at room temperature, while samples were constantly rotated and quenched by the addition of 125 mM glycine with subsequent incubation at room temperature for 5 min. The plant extract was centrifuged for 20 min at 20,000× *g* and 4 °C to remove insoluble plant material. An aliqout of the supernatant was used for a BCA assay (Pierce™ BCA™ Protein-Assay), in order to normalize the input amount used for immunoprecipitation according to protein content. The rest of the supernatant was stored at −80 °C.

For the DO-RIP, 8 µL affinity-purified anti-CP31A antibody [19] was bound to 50 µL Dynabeads ProteinG (Invitrogen) under rotation (15 rpm). The plant extract was thawed and centrifuged for 10 min at 20,000× *g* and 4 °C. A volume representing approximately 2 mg of protein of each sample was filled up to 790 µL using CO-IP buffer (150 mM NaCl, 20 mM Tris-HCl pH 7.5, 2 mM MgCl_2_, 0.5% Nonidet P-40, 5µg/mL Aprotinin). 10 µL RNaseI (0.5U/µL) were added to each sample, as well as the antibody-coated magnetic beads resuspended in 200 µL CO-IP buffer. The samples were then incubated for 60 min at 15 rpm and 4 °C. A total of 190 µL of the antibody-bead solution was taken as the input sample. After the IP, the beads were washed four times in CoIP buffer and resuspended in Proteinase K buffer (100 mM NaCl, 10 mM Tris-HCl pH 7.0, 1 mM EDTA, 0.5% SDS). 10 µL SDS (10%) were added to the input samples.

The crosslink was reversed with 0.1 mg/mL Proteinase K (ThermoFisher Scientific, Darmstadt, Germany) at 50 °C for 1 h and RNA was extracted from input and pellet fractions using TRIzol and RNA Clean and Concentrator Columns (Zymo Research, Irvine, CA, USA) according to the manufacturer’s instructions.

Library preparation was performed with the NEBNext^®^ Multiplex Small RNA Library Prep Set for Illumina (New England BioLabs, Frankfurt, Germany) according to the manufacturer’s instructions with few deviations. Library preparation was performed for half the volume. Additionally, a 5′ adaptor including unique molecular identifiers (UMI) was used (5′-rGrUrUrCrArGrArGrUrUrCrUrArCrArGrUrCrCrGrArCrGrArUrCGATCNNNNNNNN-3′). PCR amplification was performed using the KAPA HiFi HotStart ReadyMix with the cycling protocol for library amplification for Illumina platforms and an annealing temperature of 62 °C. The PCR amplified cDNA construct was purified using the GeneJET PCR Purification Kit (ThermoFisher Scientific) according to the manufacturer’s instructions and then separated on 6% polyacrylamide gel. Library fragments between 160 bp and 190 bp were extracted from the gel according to the NEBNext^®^ protocol and subjected to Illumina sequencing.

For the bioinformatic identification of the CP31A target RNAs, the following steps were performed. The samples were adapter trimmed and filtered for reads size between 40 and 60 bp, using TrimGalore (0.6.4). The 8 bp UMI sequence in the RNA adapter was extracted and reads were deduplicated after mapping using umi_tools (0.5.4). Mapping was performed using STAR (2.5.3). The *Arabidopsis thaliana* genome TAIR 10 was used as reference. The intron length was set to minimum 500 bp and maximum 1200 bp, reflecting the intron length in the chloroplast genome. Read counts were calculated using the featureCounts function of Rsubread (2.0.1) for all protein-coding genes in the chloroplast, including intronic sequences and 100 bp of upstream and downstream UTR. Read counts of all samples were normalized by gene length and library depth using TPM. In order to avoid biased results from stochastic noise, a high pass filter was used to exclude genes from the analysis, which had less than 5 read counts in any sample. The TPM scores for each gene were log2-transformed for the calculation of enrichment values of pulldown samples over input samples and for the calculation of significance of enrichment by a one-sided, paired t-test using the three replicates of input and pulldown samples.

### 4.4. Microarray Analysis

Leaves from two-weeks-old *Arabidopsis thaliana* WT and *cp31a* seedlings (1.6 g each) were homogenized in liquid nitrogen, thawed in 10.6 mL ribosome extraction buffer [46] and flash-frozen in liquid nitrogen. Total RNA was extracted using TRIzol^TM^ (Thermo Fisher) according to the manufacturer’s instructions. DNA was removed by multiple DNaseI treatments. Ribosomal RNA (rRNA) was removed from the samples using the Plant Leaf Ribo-Zero^TM^ Magnetic Kit (Illumina Inc., San Diego, CA, USA) according to the manufacturer’s instructions. The rRNA-depleted RNA was fragmented by incubation in fragmentation buffer [46] for 12.5 min at 85 °C. The reaction was stopped by the addition of EDTA, pH 8.0 to a final concentration of 50 mM. RNA was extracted using phenol/chloroform/isoamyl alcohol and ethanol precipitation. Equal amounts of fragmented RNA from WT and *cp31a* were labeled with fluorescent dyes (Cy5 and Cy3) using the ULS^TM^ aRNA labeling kit (KREATECH Diagnostics, Leica Biosystems, Nussloch, Germany) according to the manufacturer’s instructions. Hybridization was performed as in [46] to a custom microarray (MYcroarray, Ann Arbor, Mi, USA) covering the complete *Arabidopsis thaliana* chloroplast genome. Array washing, scanning and initial analysis was performed as described in [47]. Probes were assigned to different gene categories (see Supplementary Table S3). Median fluorescent ratios of WT versus *cp31a* mutants were normalized to their total intensity between the three biological replicates. Normalized median ratios were used to calculate the mean of all replicates. Probes with less than six spots total, probes overlapping genes belonging to different categories and rRNA probes were not included in the analysis. Only exon probes showing at least 1.5-fold stronger signals in WT versus *cp31a* mutants were scored.

### 4.5. RNA Extraction and RNA Gel Blot Analysis

Total RNA was extracted from fully developed leafs (0.1 g) powdered in liquid nitrogen using Trizol (Thermo Fisher) according to the manufacturer’s protocol. DNA was removed from RNA samples by three consecutive DNase I treatments and Phenol/Chloroform/Isoamyl alcohol extractions. Total RNA (4 µg) was fractioned on 1.2% agarose gels containing 1.2% formaldehyde, blotted and hybridized with radiolabeled RNA probes produced by T7 in vitro transcription from PCR products generated with primer combinations described in Supplementary Table S4.

### 4.6. Analysis of NDH Complex Abundance

Thylakoids were isolated as described in [48] from four-week-old plants grown on potting soil (A210, Stender AG, Schermbeck, Germany) exposed to a long-day light regime (80–120 µE m^2^ s^−1^, 16 h light/8 h dark) in a temperature-controlled growth cabinet (22 °C in the light phase, 18 °C in the dark phase). Chlorophyll concentration was determined according to [49]. SDS-, BN-PAGE and immunodetection assays were carried out as described [50]. NdhT signals derived from PSI-NDH supercomplexes were quantified with the Bio-1D software (Vilbert Lourmat, Eberhardzell, Germany).

### 4.7. NDH Complex Activity Analysis

The postillumination chlorophyll fluorescence rise was determined as described in Shikanai et al. 1998 using an imaging chlorophyll fluorometer (Imaging-PAM system; Heinz Walz GmbH, Effeltrich, Germany). After dark-incubation for 20 min, seedlings in the four-leaf stage were exposed to pulsed, blue measuring light. Maximal chlorophyll fluorescence (Fm) was recorded by applying a short saturating light flash. Then, plants were subjected to 5 min of blue actinic light (80 µmol × m^−2^ s^−1^) followed by dark incubation to analyse the postillumination chlorophyll fluorescence rise of cotyledons and primary leaves. CET activity measurements of ruptured chloroplasts were carried out as described [51,52].

## Supplementary Materials

Supplementary materials can be found at https://www.mdpi.com/1422-0067/21/16/5633/s1.

## Abbreviations

RBP	RNA binding protein
DO-RIP-Seq	Digestion-optimized RNA co-immunoprecipitation and sequencing
cpRNP	Chloroplast ribonucleoprotein
NDH complex	NAD(P)H dehydrogenase-like (NDH) complex
Fd	ferredoxin
