# Supplementary material for: The Chloroplast RNA Binding Protein CP31A Has a Preference for mRNAs Encoding the Subunits of the Chloroplast NAD(P)H Dehydrogenase Complex and Is Required for Their Accumulation
Source: Int J Mol Sci. 2020 Aug 6;21(16):5633. doi: 10.3390/ijms21165633 (PMC7460601; doi:10.3390/ijms21165633)
Supplement: Supplementary file 1 [file ijms-21-05633-s001.zip › Suppl. Submission/FigS2newv2.pdf]

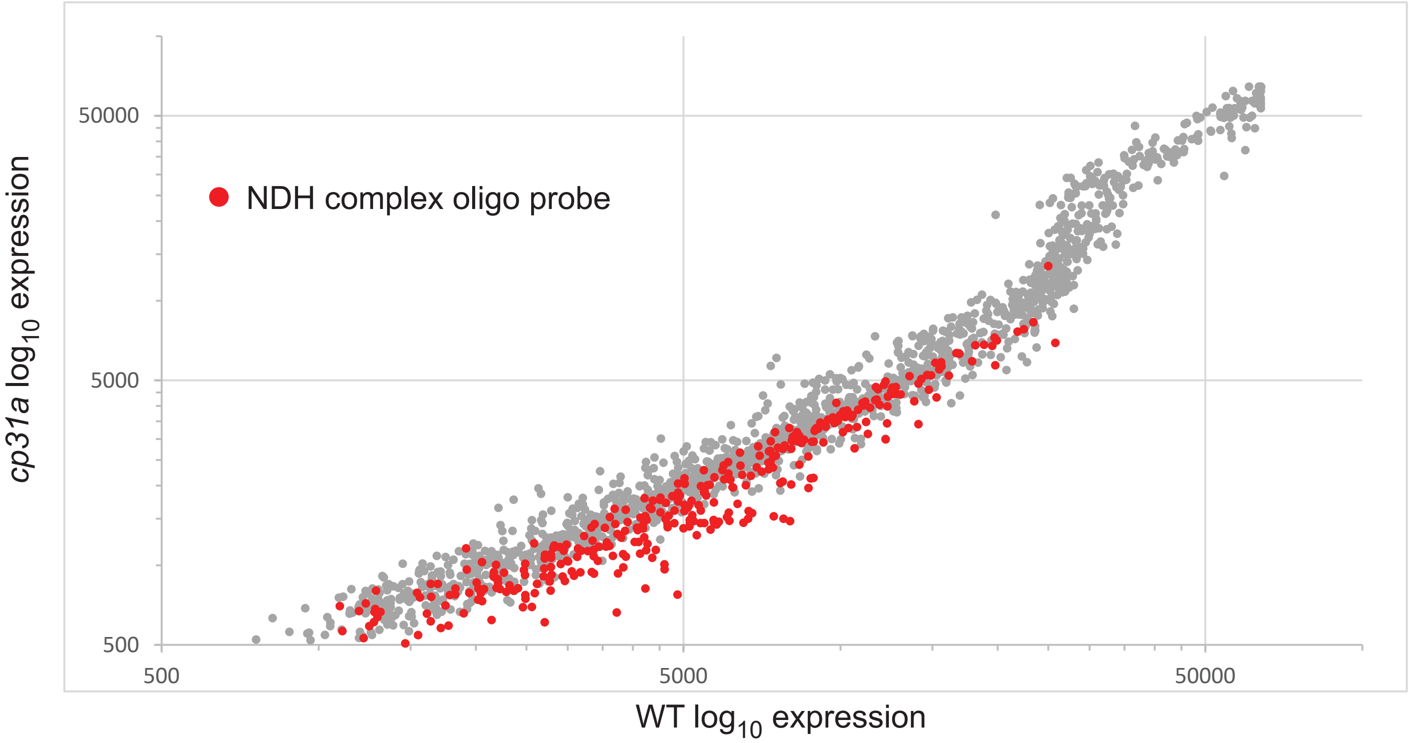

Supplemental Figure 2. Scatter plot for DNA microarray analysis of chloroplast gene expression affected by the null mutation of the *cp31a* gene. The scatter plot shows the averages of normalized signal means from the wt strain and those from the *cp31a* mutant (n = 2) for each oligonucleotide probe on the array. The red dots indicate probes that represent *ndh* gene sequences. Note that the *ndh* probes are found at the lower edge of overall probe distribution, which indicates reduced RNA levels relativ to other probes.
