# Supplementary material for: The Chloroplast RNA Binding Protein CP31A Has a Preference for mRNAs Encoding the Subunits of the Chloroplast NAD(P)H Dehydrogenase Complex and Is Required for Their Accumulation
Source: Int J Mol Sci. 2020 Aug 6;21(16):5633. doi: 10.3390/ijms21165633 (PMC7460601; doi:10.3390/ijms21165633)
Supplement: Supplementary file 1 [file ijms-21-05633-s001.zip › Suppl. Submission/Figure S1.pdf]

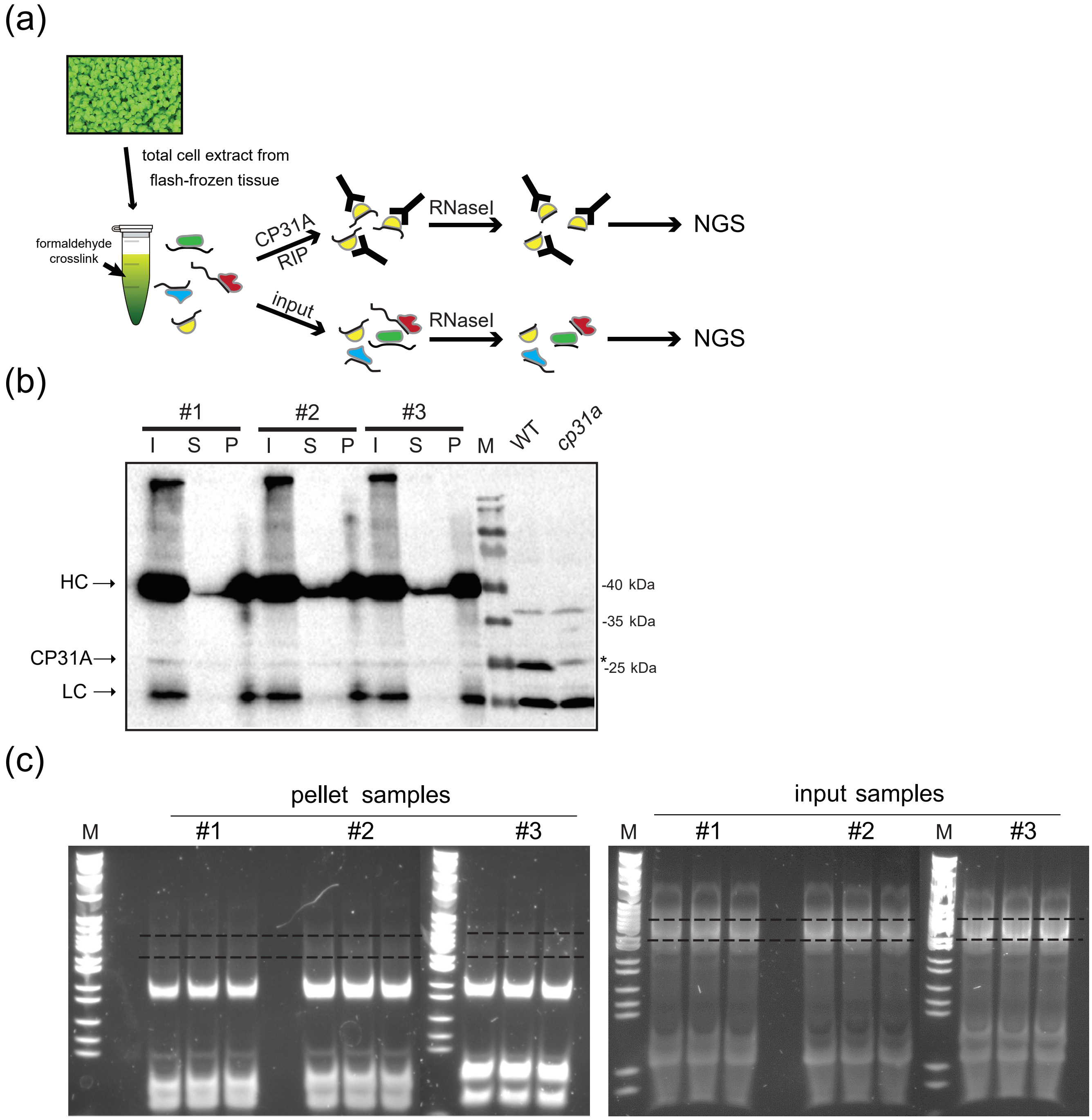

Supplemental Figure 1. DO-RIP-seq analysis. (**a**) Schematic overview of DO-RIP-Seq. Seedlings are grown for 14 days prior to flash-freezing and whole cell lysate preparation. Lysates are cross-linked with formaldehyde, digested with RNaseI, and RNA binding proteins (here cpRNPs) are immunoprecipitated. RNAs from precipitates and from the input were analysed by next generation sequencing (NGS). (**b**) Immunoblots of DO-RIP-fractions from WT plants. The blots were probed with the affinity-purified CP31A antibody used in the DO-RIP-Seq assays. A band representing a cross-reacting protein is found in *cp31a* mutants slightly larger than CP31A itself (asterisk). IgGs in the immunoprecipitates (HC = heavy chain; LC = light chain) are detected by the secondary antibody used to probe the immunoblot. P, S, and I = pellet, supernatant and input fractions of DO-RIP experiments. M = Molecular mass marker. (**c**) Purified DO-RIP-seq libraries from pellet and input fractions were separated on a 6% Polyacrylamide gel for size-selection. The gel was stained with SYBR Gold nucleic acid gel stain and visualized on a UV transilluminator. Left: Gel with pellet samples prior to size selection. Right: Gel with input samples prior to size selection. The area excised from the gel for library purification is indicated by black, dashed bars. This area corresponds to adapter-ligated constructs derived from around 30 to 70 nucleotide RNA fragments. M = molecular mass marker.
