# Supplementary material for: The Chloroplast RNA Binding Protein CP31A Has a Preference for mRNAs Encoding the Subunits of the Chloroplast NAD(P)H Dehydrogenase Complex and Is Required for Their Accumulation
Source: Int J Mol Sci. 2020 Aug 6;21(16):5633. doi: 10.3390/ijms21165633 (PMC7460601; doi:10.3390/ijms21165633)
Supplement: Supplementary file 1 [file ijms-21-05633-s001.zip › Suppl. Submission/Table S1.pdf]

Supplemental Table 1: Quality analysis of replicate experiment in Do-RIP-Seq assays using the Pearson correlation

	**I-1**	**I-2**	**I-3**	**P-1**	**P-2**	**P-3**
**I-1**	1.000					
**I-2**	0.978	1.000				
**I-3**	0.980	0.998	1.000			
**P-1**	0.963	0.993	0.991	1.000		
**P-2**	0.961	0.992	0.990	0.999	1.000	
**P-3**	0.963	0.992	0.990	0.999	1.000	1.000

Numbers in the table refer to the Pearson correlation coefficients between DO-RIP-seq samples. Correlations were calculated using DESeq2 and R stats package. Read counts were calculated for all chloroplast genes using summarizeOverlaps in 'Union' mode. The counts were then normalized by 'regularized log' transformation. Normalized counts were then used as input for 'cor' function using the Pearson method. I = input; P = pellet; numbers refer to numbers of replicates
