# Supplementary material for: The Chloroplast RNA Binding Protein CP31A Has a Preference for mRNAs Encoding the Subunits of the Chloroplast NAD(P)H Dehydrogenase Complex and Is Required for Their Accumulation
Source: Int J Mol Sci. 2020 Aug 6;21(16):5633. doi: 10.3390/ijms21165633 (PMC7460601; doi:10.3390/ijms21165633)
Supplement: Supplementary file 1 [file ijms-21-05633-s001.zip › Suppl. Submission/Table S4.pdf]

Supplemental Table 4: Oligonucleotides

Oligonucleotide	Sequence (5'>3')	Target(s)	Purpose
ndhBex1.rp	CCGATGGAGAGAAGAACCTATG	*ndhB*	RNA gel blot analysis
ndhBex1.T7	GTAATCGACTCACTATAGGGTAGCCAAGAGAAACCATGAA		RNA gel blot analysis
ndhD.rp	CATGTGGGGTGGAAAGAAAC	*ndhD*	RNA gel blot analysis
ndhD.T7	GTAATCGACTCACTATAGGGATTACGTTGACCGGGAGATG		RNA gel blot analysis
ndhF_short.T7	TAATACGACTCACTATAGGGAGAAGAGATGCGACTTCCAC	*ndhF*	RNA gel blot analysis
ndhF_short.rp	TTTTTCACGCCGTCAATAAACC		RNA gel blot analysis
ndhK.rp	CTATGGCCGCTTCTTTATGG	*ndhK*	RNA gel blot analysis
ndhK.T7	GTAATCGACTCACTATAGGGATTTCCAATATGCGGACTGC		RNA gel blot analysis
